# Pericytes of Indirect Contact Coculture Decrease Integrity of Inner Blood-Retina Barrier Model *In Vitro* by Upgrading MMP-2/9 Activity

**DOI:** 10.1155/2021/7124835

**Published:** 2021-09-29

**Authors:** Tianye Yang, Liang Guo, Yuan Fang, Mingli Liang, Yongzheng Zheng, Mingdong Pan, Chun Meng, Guanghui Liu

**Affiliations:** ^1^Department of Bioengineering, College of Biological Science and Biotechnology, Fuzhou University, Fuzhou, Fujian, China; ^2^Lunan Pharmaceutical Group Co. Ltd., Linyi, Shandong, China; ^3^Department of Ophthalmology, Affiliated People's Hospital (Fujian Provincial People's Hospital), Fujian University of Traditional Chinese Medicine, Fuzhou, Fujian, China; ^4^Eye Institute of Integrated Chinese and Western Medicine, Fujian University of Traditional Chinese Medicine, Fuzhou, Fujian, China

## Abstract

Inner blood-retina barrier (iBRB) is primarily formed of retinal microvascular endothelial cells (ECs) with tight junctions, which are surrounded and supported by retinal microvascular pericytes (RMPs) and basement membrane. Pericytes are believed to be critically involved in the physiology and pathology of iBRB. However, the underlying mechanism remains to be fully elucidated. We developed a novel *in vitro* iBRB model which was composed of primary cultures of rat retinal ECs and RMPs based on Transwell system. We tested the involvement of pericytes in the migration and invasion of ECs, examined the expression and activity of matrix metalloproteinase- (MMP-) 2/MMP-9 in the culture, evaluated the TEER and permeability of iBRB, and assessed the expression of ZO-1, occludin, claudin-5, and VE-cadherin of endothelial junctions. We found that RMPs with indirect contact of ECs can increase the expression of MMP-2 and upgrade the activity of MMP-2/9 in the coculture, which subsequently decreased TJ protein abundance of ZO-1 and occludin in ECs, promoted the migration of ECs, and finally reduced the integrity of iBRB. Taken together, our data show that RMP relative location with ECs is involved in the integrity of iBRB via MMP-2/9 and has important implications for treating diabetic retinopathy and other retinal disorders involving iBRB dysfunction.

## 1. Introduction

Inner blood-retina barrier (iBRB), mainly consisting of endothelial cells (ECs), retinal microvascular pericytes (RMPs), and basement membrane, plays a key role in retinal homeostasis [1]. Under physiological conditions, blood-retina barrier (BRB) restricts nonspecific transport between neural retina and peripheral blood and prevents the passage of pathogens, toxins, toxicants, proteins, and neurotransmitters into the retina [2]. Under pathological conditions, such as hyperglycemia, hypoxia, oxidative stress, or inflammation, disruption of integrity of interendothelial junctions leads to the increase of iBRB permeability and gathering of harmful substances in the retina [3].

The barrier function is exerted primarily by the EC layer. The sophisticated junctions between adjacent ECs, including tight junctions (TJs), adherens junctions (AJs), gap junctions, and desmosomes, are the basis of iBRB [4]. Besides their involvement in cell to cell adhesion, these structures sustain cell survival, cell polarity, and paracellular permeability [5]. TJs, forming a paracellular diffusion barrier to limit the movement of molecules across EC layer, take a central role in the maintenance [4]. It has previously been demonstrated that increasing of iBRB permeability is related with the degradation of TJs of ECs *in vitro* and *in vivo* [5, 6]. Thus, the regulation of iBRB permeability is critical both for protecting the retina from harmful components of peripheral blood and for the treatment of retinal disorders.

iBRB is regulated principally by interactions among ECs, pericytes, and extracellular matrix (ECM). Pericytes are located in the outer wall of EC tubes [7]. As part of the microvasculature, pericytes maintain vascular stability and enhance endothelial barrier function by direct contact and paracrine regulation [1, 8], while pericytes detached from ECs would lead to unstabilization of endothelial barrier [5], decrease in transendothelial electrical resistance (TEER), and increase in permeability [9]. Due to multiple effects on ECs, the role of RMPs in iBRB should be further explored. Basement membrane, a specialized ECM of retinal microvessel, encloses ECs and pericytes and provides the intermediates for cell communication. The molecular components of basement membrane, secreted by the neighboring ECs and pericytes, act as a crucial clue for appropriate TJ assembly and iBRB properties to maintain function of mature iBRB [10, 11].

Matrix metalloproteinases (MMPs), one of the major mediators of barrier degradation, modulate the structure and function of ECM molecules under physiological and pathological processes [12–14]. The involvement of MMPs in iBRB disruption during diabetic retinopathy, retinal ischemia, and retinal neovascularization has been demonstrated in numerous studies [15–17]. The association between the expression levels of different MMPs and the presence of ECs and pericytes has also been suggested [18], but it has not been fully elucidated.

MMP-2/9, members of the most ubiquitous members of MMPs family, are not only able to degrade ECM protein but also able to cleave TJ proteins [19]. They are known as mediators of endothelial barrier disruption and TJ protein proteolysis [20]. MMP-2/9 can be secreted both by ECs and pericytes [18, 21–23]. MMP-2 activity can increase when ECs are cocultured with pericytes [24].

Here, we used an *in vitro* iBRB model system composed of primary cultures of rat ECs and RMPs, studied the expression and activity of MMP-2/9 secreted by ECs and RMPs under indirect contact coculture, and explored the effect of MMP-2/9 on the maintenance of iBRB function *in vitro*. We for the first time provide evidence demonstrating that indirect EC-RMP cell to cell interaction increases MMP-2 expression, upgrades MMP-2/9 activity, disrupts TJs of ECs, and decreases the integrity of iBRB *in vitro*. This evidence is pivotal for further understanding RMP modulation of iBRB properties with respect to the future development of specific strategies for treatment of certain retina diseases or pathological conditions with iBRB involvement due to MMP imbalance.

## 2. Materials and Methods

### 2.1. Preparation and Cultivation of Rat Retinal ECs and RMPs

All experimental procedures were conducted in accordance with the ARVO Statement for the Use of Animals in Ophthalmic and Vision Research and approved by the Institutional Ethics Committee for Animal Use in Research and Education at Fuzhou University (Fujian, China).

The retinas for primary retinal cell culture were isolated from 3-week-old male rats (Sprague Dawley rat; SLAC, Shanghai, China). The rat retinal ECs and RMPs were obtained and identified as reported [25, 26]. Three-dimensional (3D) capillary-tube formation assay was used to assess the function of ECs and RMPs *in vitro* as previously described [26]. Briefly, ECs and RMPs were labeled by cell tracker (Molecular Probes, OR, USA) before 3D coculture in Matrigel (BD Biosciences, CA, USA). After 24 hours of coculture, the cells were evaluated by fluorescent microscopy (Nikon, Tokyo, Japan). The cells of the 3^rd^-7^th^ passage were used for further experiment after they were proved being functional cells by the assay.

### 2.2. *In Vitro* iBRB Model

**Figure 1:**
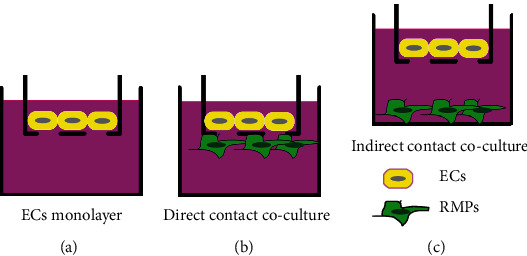
Schematic diagrams of iBRB models *in vitro*. (a) Model ECs. EC monolayer without RMPs. (b) Model DC. Coculture of ECs directly contacted with RMPs. (c) Model IDC. Coculture of ECs indirectly contacted with RMPs.

Three basic *in vitro* iBRB models, shown as the schematic description, were designed based on Transwell system (0.4 *μ*m pores, Corning, NY, USA). The EC monolayer (model ECs) was made by ECs alone (Figure 1(a)). The direct contact coculture (model DC) was made through ECs directly cocultureing with RPMs cells (Figure 1(b)). The indirect contact coculture (model IDC) was made by ECs and RMPs with indirect contact (Figure 1(c)). Transwell system allows cells of coculture to communicate directly or indirectly through the pores of the polyethylene terephthalate membrane. For establishing model DC, RMPs and ECs were layered separately on each side of a 24-well Transwell membrane. RMPs were seeded on the outside of the membrane when the Transwell insert was inverted. 12 h later, when RMPs adhered to the membrane, the insert with RMPs was placed in well of a 24-well plate (Corning, NY, USA), and ECs were seeded on the inner side of the membrane. For establishing model IDC, Transwell inserts with EC monolayer were placed into 24-well plates culturing with 80% confluent layers of RMPs. All iBRB models were seeded with ECs or RMPs at the same passage number and density (6 × 10^4^ cells/mL). The cells were grown on the inserts for 48 h before being used in each experiment.

### 2.3. Migration Assay and Invasion Assay

The ability of cells to migrate was assessed by scratch wound healing assay. The cells (ECs or ECs mixed with RMPs) were seeded onto 24-well plates and cultured to confluence. A scratch was made by a 10 *μ*l sterile pipette tip in the monolayer perpendicularly across the center of the well. The floating cells were washed away with serum-free cell culture media. The wound closure was visualized by time-lapse imaging with a phase-contrast microscope (Nikon, Tokyo, Japan). Phase-contrast images of five selected fields were acquired at 0, 24, and 32 h. The area of the gap that has not been covered by the cells was analyzed using the ImageJ software (ImageJ 1.8.0; National Institutes of Health, MD, USA).

The invasive ability of cells was evaluated using Transwell system (8 *μ*m pore size, Corning, NY, USA). The Transwell chambers were coated with 0.1 mL Matrigel (50 *μ*g/mL) and put into the 24-well plates. EC suspension (1 × 10^5^ cells) was seeded to the upper chamber of Transwell. RMP suspension (1 × 10^5^ cells) was added to the lower chamber of 24-well plates. 10% serum growth media was added to the chamber, and the cells were allowed to invade for 32 hours at 37°C in a 5% CO_2_ humidified incubator. Then, the cells were fixed with 20% methanol and stained with 0.5% crystal violet. The cells on the top surface of the filter were removed by a cotton swab, and the cells that had invaded into the bottom surface of the filter were imaged and counted under with phase-contrast microscope over five random fields of each well.

### 2.4. Zymography

The activity of MMP-2/9 was estimated by substrate gelatin zymography according to George et al. [27]. Supernatants of the cell culture were collected and mixed with 5x sample buffer (10% glycerinum, 10% SDS, 1% bromophenol blue, 500 mM Tris–HCl, pH 6.8), and equal sample volumes were loaded on 10% SDS polyacrylamide gels which contained 0.1% gelatin (Sigma, MO, USA). After the electrophoresis step, the gels were treated twice with 2.7% Triton-X 100 solution for 30 min each and then incubated in developing buffer (50 mM Tris, 200 mM NaCl, 5 mM CaCl_2_, and 1 mM ZnCl, pH 7.5) for 20 h at 37°C. After the incubation, the gels were stained in staining solution (25% alcohol, 10% acetic acid, and 0.25% coomassie blue) for 60 min and then treated with destaining solution (25% alcohol and 10% acetic acid) for 1 h. The densitometric analysis was performed using the ImageJ software.

### 2.5. Transendothelial Electrical Resistance Measurements

Transendothelial electrical resistance measurements were performed once endothelial cultures of 3 models reached 95%-100% confluent. In Transwell (0.4 *μ*m pore size) cultures, TEER was conducted by a Millicell device (Millicell-ERS-2, MA, USA) and chopstick-like electrodes as described elsewhere [28]. One electrode was immersed in the upper chamber of Transwell and the other electrode in the lower chamber of a 24-well plate. An equilibration period at room temperature for 20 min was performed prior to the measurement. TEER value was calculated as the resistance (*Ω*) of cell culture inserts minus background resistance (*Ω*) of cell-free inserts. The difference was multiplied with the area of insert (0.33cm^2^), resulting in a TEER value given as a mean in *Ω*·cm^2^.

### 2.6. Sodium Fluorescein Permeability Measurement

Permeability measurement was assessed by sodium fluorescein since ECs form a barrier against the free diffusion of sodium fluorescein. Sodium fluorescein (100 *μ*g/mL) was added to the apical side of Transwell filter. Permeability of the cell layer to sodium fluorescein was measured according to previous report [29]. Briefly, sodium fluorescein permeates through the monolayer into the basolateral chamber. The amount of sodium fluorescein accumulating in the basolateral chamber is an indicator of the permeability of cell layer. Permeability of the cell layer was examined as fluorescence with a Varioskan LUX Multimode Microplate Reader (Thermo Scientific, MA, USA) at excitation wavelengths of 440 nm and emission wavelengths of 525 nm. GM6001 (Selleckcn, NY, USA), a broad inhibitor, was added in the medium of model IDC as comparison.

### 2.7. Western Blotting

Total proteins were isolated from the culture using RIPA lysis buffer (#P0013B, Beyotime, Jiangsu, China) mixed with protease inhibitor and PMSF. SDS-PAGE was used to separate the proteins. The separated proteins were transferred to a PVDF membrane (#88520, Millipore, MA, USA), which was blocked with 5% bovine serum albumin at room temperature for 2 h and then immunoblotted with antibodies against MMP-9 (#ab228402, Abcam, MA, USA), MMP-2 (#ab86607, Abcam, MA, USA), zonula occludens-1 (ZO-1, #ab96587, Abcam, MA, USA), occludin (#ab216327, Abcam, MA, USA), claudin-5 (#ab131259, Abcam, MA, USA), and vascular endothelial- (VE-) cadherin (#ab33168, Abcam, MA, USA). Chemiluminescence was detected with the ChemiDoc MP imager (Bio-Rad, CA, USA). *β*-Actin was used as a negative control, and the results were normalized to *β-*actin.

### 2.8. Statistical Analyses

All experiments were repeated at least three times. Statistical analyses were conducted using the JASP software package (Version 0.14.1, JASP Team, Amsterdam, Netherlands). Student's *t*-test was used to determine significant differences between two groups, and one-way ANOVA with the least significant difference test was performed to compare more than two groups. All data are presented as mean ± SD, and a value of *P* < 0.05 was considered statistically significant.

## 3. Results

### 3.1. RMPs Inhibited EC Migration in Direct Contact Coculture and Prompted EC Migration in Indirect Contact Coculture

We used 2D culture to evaluate EC migration with the involvement of pericytes at the first step. In a wound-induced migration assay, the confluent cell monolayer was disrupted and cells were allowed to move into the cell-free area.

**Figure 2:**
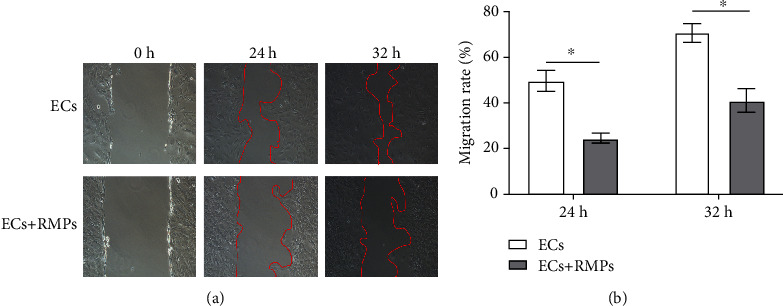
RMPs inhibit EC migration in direct contact coculture. (a) Typical pictures of wound-induced migration assay. Wound repair 24 h and 32 h after the mechanical scratch (top, ECs cultured alone; bottom, ECs cocultured with RMPs). The red lines indicate the edges of wounded area. (b) Quantitative analysis of wound repair 24 h and 32 h after making the scratch. Initial wound area at 0 h is defined as 100%. Migration rate = (1 − wound area at a specific time point/initial wound area)∗100%. Analytical data are presented as the mean ± SD. ^∗^*P* < 0.01.

The mobility of ECs cocultured with RMPs was decreased in the scrape-wound assay as compared with ECs cultured alone (Figure 2(a)). The scratched areas of ECs cultured alone were almost fully repopulated in 32 h, whereas migration of ECs cocultured was greatly halted by RMPs. A decrease of 41.6% in inhibition of migration was observed in the coculture compared with that in the culture of ECs alone in 32 h (Figure 2(b)). It indicated the involvement of pericytes in the stability of ECs under condition of direct contact with each other.

**Figure 3:**
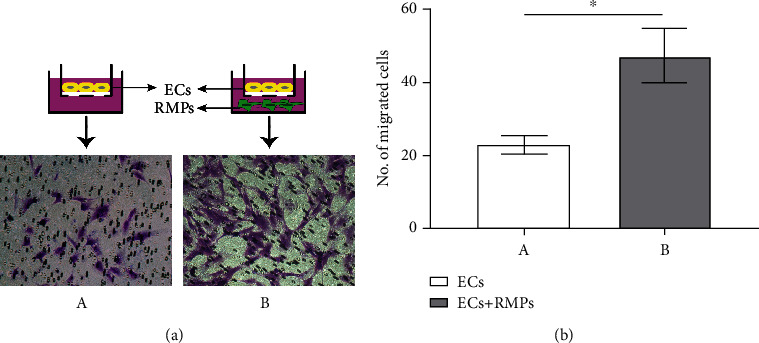
RMPs promote EC migration in indirect contact coculture. (a) EC suspensions are layered onto Matrigel-coated Transwell inserts, and RMP suspensions are layered onto the bottom of 24-well plates. RMPs promote invasion of ECs through Matrigel in invasion assays. (b) Number of cells traversing the insert is calculated as a measure of invasion. Analytical data are presented as the mean ± SD. ^∗^*P* < 0.01.

ECs have to migrate and cross basement membrane before new vessel formation. To invade through basement membrane, ECs must degrade the components of it. We used 3D indirect contact coculture model to assess the ability of pericytes to modulate EC invasion in Matrigel invasion assays secondly. Significant difference in invasiveness was noted between the indirectly contact cocultured cells and ECs cultured alone in the assay. The invasive potential of ECs in indirect contact coculture was substantially increased (Figure 3(a)). In the Matrigel assay, quantitative analysis of invading cells showed that the invasiveness of indirectly contact cocultured ECs was greatly increased to 45% as compared with the ECs cultured alone (Figure 3(b)). It suggests that pericytes have a promoting effect on the invasion of ECs under indirect contact condition.

### 3.2. RMPs in Indirect Contact Coculture with ECs Upgraded the Expression of MMP-2 and Activity of MMP-2/9 in ECs

MMP-2/9 are the major mediators of basement membrane degradation and the key contributors to the invasion of ECs through basement membrane. To determine the relationship between RMP relative location and EC invasion and address the impact of RMPs on MMP expression of iBRB model *in vitro*, MMP-2/9 are evaluated. We performed Western blot and gelatin zymographical analysis of the culture and explored the secretion and activity of MMP-2/9.

**Figure 4:**
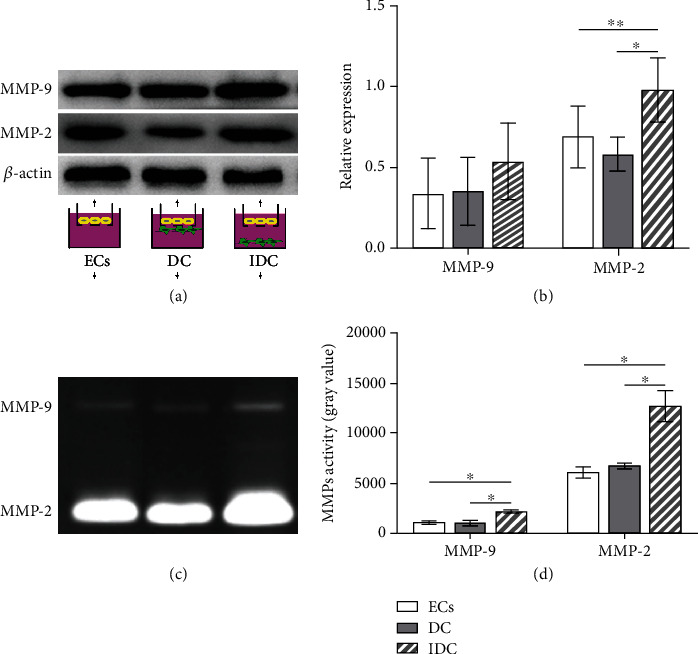
RMPs upgrade expression of MMP-2 and activity of MMP-2/9 of indirect contact coculture. (a) The abundance of MMP-9 and MMP-2 is confirmed via Western blot in model ECs, model DC, and model IDC. (b) MMP-9 and MMP-2 expression measurements are quantified via normalization to *β*-actin. (c) Activity of MMP-9 and MMP-2 is evaluated by zymography in 3 models. (d) Gray value of MMP-9 and MMP-2 activity measurements. ^∗^*P* < 0.01 and ^∗∗^*P* < 0.05.

Indirectly contact cocultured cells (model IDC) secreted significantly more MMP-2 than ECs cultured alone (model ECs) or direct contact coculture (model DC), but no difference was observed between ECs cultured alone and direct contact coculture. No difference of MMP-9 expression was shown among the 3 iBRB models (Figures 4(a) and 4(b)). Additionally, we observed that MMP-2/9 activity of indirectly contact cocultured cells by zymography was higher than that of ECs cultured alone and direct contact coculture (Figures 4(c) and 4(d)). These observations suggest that pericyte metabolites may enhance the expression of MMP-2 and the activity of MMP-2/9 to promote the invasion of ECs under the condition with indirect contact.

### 3.3. RMPs Downgraded TEER and Upgraded Permeability of iBRB under the Condition with Indirect Contact Coculture

**Figure 5:**
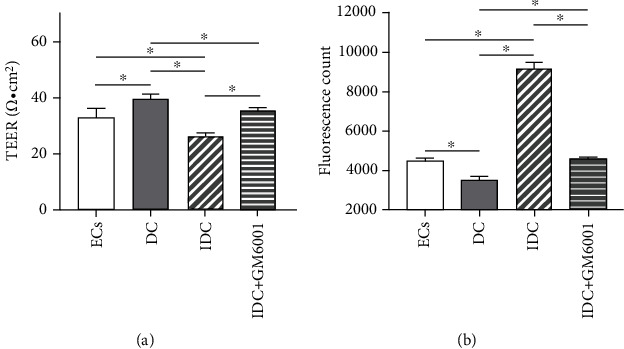
RMPs downgrade TEER and upgraded permeability of iBRB under the condition with indirect contact. (a) Value of TEER in model ECs, model DC, model IDC, and model IDC+GM6001. (b) Permeability of sodium fluorescein in model ECs, model DC, model IDC, and model IDC+GM6001. ^∗^*P* < 0.01. Unit = arbitrary unit (A.U.).

Immigration of ECs occurs before the degradation of iBRB integrity, such as decrease of TEER or increase of permeability. TEER and permeability of iBRB models were evaluated and presented in Figure 5. TEER of model ECs was 33.00 ± 3.30 *Ω* · cm^2^ and that of model DC was 39.60 ± 1.65 *Ω* · cm^2^. TEER was significantly lower in model IDC (26.18 ± 1.16 *Ω* · cm^2^) than in model ECs and model DC. To clarify the role of RMPs in the BBB disruption involved with MMP-2/9, GM6001, a broad inhibitor, was added in the medium of model IDC. However, GM6001 significantly increased the value of TEER (35.64 ± 1.51 *Ω* · cm^2^) of model IDC (Figure 5(a)).

Opposite changes were found in sodium fluorescein permeability measurement. Permeability of sodium fluorescein is taken as the marker of paracellular permeation. Permeability of model ECs was 4500 ± 200 A.U. and that of model DC was 3500 ± 200 A.U. while RMPs can increase the permeability of sodium fluorescein in the coculture with indirect contact. The permeability value of model IDC (9233 ± 305.5 A.U.) was significantly higher than that of model ECs and model DC. GM6001 significantly decreased the permeability value of ECs to 4600 ± 100 A.U. in model IDC (Figure 5(b)).

Collectively, these observations suggest that RMPs may regulate iBRB TEER and permeability by MMP-2/9 under the condition with indirect contact coculture since the application of inhibitor GM6001 changes the TEER and permeability values of EC/RMP coculture with indirect contact (model IDC).

### 3.4. RMPs Decreased the Expression of ZO-1 and Occludin of iBRB under the Condition with Indirect Contact Coculture

It has been generally accepted that TJ proteins and adhesion molecules are in correlation with integrity of iBRB, and the junction proteins get disrupted when ECs start to migrate [4]. Thus, we investigated further whether RMPs may increase iBRB permeability by disrupting expression of the TJ proteins ZO-1, occludin, and claudin-5 under condition of indirect contact coculture, as well as the AJ protein VE-cadherin.

**Figure 6:**
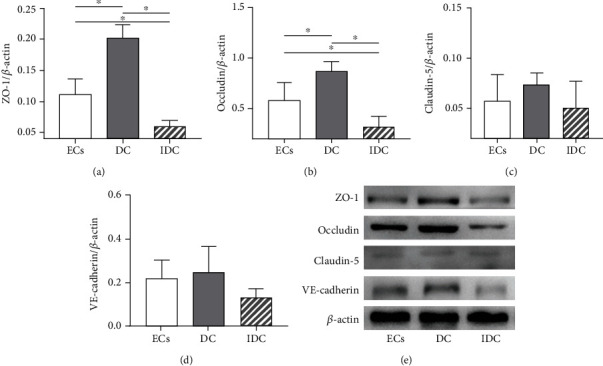
RMPs decrease the expression of ZO-1 and occludin of iBRB under the condition with indirect contact coculture. (a–d) ZO-1, occludin, claudin-5, and VE-cadherin measurements are quantified via normalization to *β*-actin. ^∗^*P* < 0.01. (e) Abundance of ZO-1, occludin, claudin-5, and VE-cadherin is evaluated via Western blot in 3 iBRB models.

Expression of ZO-1 and occludin was significantly higher in model DC than in model ECs cultured alone. RMPs or its metabolite may influence ZO-1 and occludin expression. The expression of ZO-1 and occludin was significantly lower in model IDC than in model ECs and model DC. No difference of claudin-5 and VE-cadherin expression was observed among the 3 iBRB models (Figure 6). These results suggest that RMPs may reduce the iBRB integrity under condition of indirect contact coculture by upregulating MMP-2/9 activity, which subsequently, at least in part, decrease the abundance of ZO-1 and occludin directly or indirectly.

## 4. Discussion

In this study, we developed a novel *in vitro* iBRB model system composed of primary cultures of rat retinal ECs and RMPs on the permeable membrane of Transwell. Basing on this iBRB model system, we demonstrated that RMPs prompted EC migration in indirect contact coculture associated with the increasing of MMP-2 expression and upgrading of MMP-2/9 activity. We showed that RMPs decreased the expression of ZO-1 and occludin of ECs, downgraded TEER, and upgraded permeability of iBRB in indirect contact coculture by increasing the expression of MMP-2 and activity of MMP-2/9. Our results suggest that RMP relative location has an important impact on the integrity of iBRB mediated by MMP-2/9.

Inner BRB function is exerted primarily by EC layer and supported by RMPs. Pericytes, wrap around ECs lining the capillaries, are critical components of iBRB through communication with ECs. Relative surface coverage and density of pericytes on capillaries are positively correlated with endothelial barrier properties [30]. According to the difference of vascular bed, pericyte coverage of the abluminal vessel area of ECs is partial. The retina has the highest pericyte density of all vascular beds [31], the relative frequency of RMPs to ECs is 1 : 1 [32], and the frequency of RMP coverage on retinal capillaries was reported to be high up to 94.5% [33]. Therefore, the role of RMPs in iBRB and its coordination with ECs may deserve particular attention.

Pericytes interact with ECs by foot processes directly and offer important supports to ECs, such as stabilizing the newly formed endothelial tubes and modulating EC survival, proliferation, differentiation, migration, and invasion [34]. The requirement for pericytes has been demonstrated by *in vivo* models of induced pericyte loss that lead to increased permeability of BRB [35]. In our Transwell coculture system, RMPs on the bottom side of 24-well plates can communicate with ECs on the top side of Transwell through the 0.4 *μ*m pores of the permeable membrane under indirect contact condition. Comparing to the EC monolayer cultured alone, the EC-RMP indirect contact coculture model led to an obvious migration and invasion of ECs and had more cells through the membrane of Matrigel. Interestingly, EC migration was significantly halted by RMPs in direct contact coculture (2D coculture). In previous report, it is generally considered that pericytes restrict EC migration and stabilize the blood vessels [1, 36]. Our findings suggest that RMPs may have a negative role in iBRB stability when it loses direct contact with ECs, since EC migration and invasion through basement membrane are promoted by RMPs under the condition of indirect contact coculture.

Basement membrane, a specialized ECM of microvessel and intermediate for EC-RMP communication, degrades generally with EC migration and invasion. MMPs, one of the major mediators of endothelial barrier degradation, have been shown to regulate the structure and function of ECM molecules under physiological and pathological processes [12–16]. The correlation between the expression of different MMPs and the presence of ECs and pericytes has also been suggested. Takahashi et al. reported human pericytes induced MMP-9 activity but not MMP-2 in culture [37], and the result was confirmed by Underly et al. in mouse blood-brain barrier (BBB) *in vivo* [38]. Takata et al. also reported MMP-9 derived from pericytes lead to BBB damage [39]. On the other hand, Zozulya et al. observed no difference in MMP-2 expression of different EC-pericyte coculture but an elevated expression of MMP-9 from ECs cultured in direct contact to pericytes as compared to that from ECs free of pericytes [18]. Our Wb data indicated that MMP-2 expression was elevated in indirect contact coculture compared with that in EC culture or direct contact coculture, while MMP-9 was not differentially expressed. Zymography data show that MMP-2/9 of indirect contact coculture was generally in higher activity than that of model ECs and model DC. The inconsistent observations in previous literature and our study may be owed to the differences in the type and origin of cells and the culture methods used in different studies, which also highlights the need for a better and stable model of iBRB for mechanistic investigations. Our results suggest the promotion of EC migration and invasion in indirect contact coculture by RMPs may be caused by the increase of MMP-2 expression and MMP-2/9 activity. The suggestions are further supported by the assay of TEER and sodium fluorescein permeability.

TEER, a measurement of electrical resistance across a cellular monolayer, reflects an amount of ionic molecule flux through cell layer and is a very sensitive and reliable method to confirm the integrity and permeability of the monolayer [9, 40]. Sodium fluorescein is commonly used as low-molecular-weight tracers, and permeability of sodium fluorescein represents an indication of paracellular permeation [9, 41]. Previous studies of *in vitro* coculture models have shown that addition of pericytes increases the TEER of EC monolayer [42–44]. In this study, RMPs directly cocultured with ECs had a positive role in iBRB, but RMPs indirectly contact cocultured with ECs decreased the TEER and increased the permeability of iBRB. The results are similar to the *in vitro* finding of BBB model which demonstrated pericytes can negatively regulate endothelial barrier integrity via paracrine in indirect contact coculture system [9]. Our results further revealed that MMP-2/9 mediate RMP regulation of iBRB integrity in indirect contact condition since the changes of integrity in model IDC can be inhibited by MMP broad inhibitor GM6001. But MMP-2/9-mediated RMP regulation may not be the only mechanism of RMP modulation of iBRB *in vitro* due to the incomplete inhibition of GM6001.

Generally, MMPs open endothelial barrier by degrading cellular junctions [20, 45]. TJs and AJs, both important in keeping permeability low while providing a high TEER, are specific type of cell-cell contacts that obstruct paracellular pathway for solute diffusion and regulate the paracellular passage of small molecules such as water and ions [6, 46]. TJs of ECs are mainly composed of claudins, occludin, ZO, and tight junction adhesion molecules, and VE-cadherin is the major compound of endothelial AJs [5]. Pericyte recruitment to ECs can induce TJ formation [35], while detachment of pericytes from ECs would lead to the unstabilization of EC junctions [5]. In the present study, no significant difference was observed in the expression of claudin-5 and VE-cadherin among the 3 iBRB models. The data suggest that decreasing the TJ protein's expression of ZO-1 and occludin may be the mechanism of RMP regulation on iBRB integrity under indirect contact condition. The suggestion may improve our understanding of the pathological phenomenon in diabetic retinopathy *in vivo* that the loss of RMPs or the loss of direct contacts of ECs with RMPs occurs before BRB dysfunction and EC migration. But how RMPs regulate MMP-2/9 to affect iBRB integrity when RMPs lose indirect contact with ECs remains to be elucidated. Future studies will address these limitations by conducting experiments in EC-specific MMP-2/9-KO mice, investigating the role of exosome in paracrine signals of RMPs and ECs and determining how RMPs contribute to the integrity and function of iBRB in clinical studies when the loss of direct contact of ECs with RMPs occurs.

## 5. Conclusions

In summary, the results presented here are the first to reveal the influence of RMP relative location on iBRB model *in vitro*. RMPs with indirect contact of ECs can increase expression of MMP-2 and upgrade activity of MMP-2/9, which subsequently decreases TJ protein abundance of ZO-1 and occludin in ECs, promotes the migration of ECs, and finally reduces the integrity of iBRB. Since the loss of direct contacts of ECs with RMPs is a general phenomenon in retinal pathology, our observations likely have important implications for treating diabetic retinopathy and other retinal disorders involving iBRB dysfunction.

## Data Availability

The datasets used and/or analyzed during the current study are available from the corresponding author on reasonable request.
